# Supplementary material for: Effect of Ultra-Processed Foods Consumption and Some Lifestyle Factors during Pregnancy on Baby’s Anthropometric Measurements at Birth
Source: Nutrients. 2022 Dec 22;15(1):44. doi: 10.3390/nu15010044 (PMC9824718; doi:10.3390/nu15010044)
Supplement: Supplementary file 1 [file nutrients-15-00044-s001.zip › nutrients-2027714-supplementary.pdf]

SUPPLEMENTARY MATERIAL

FIGURE S1 - Participants included in the study

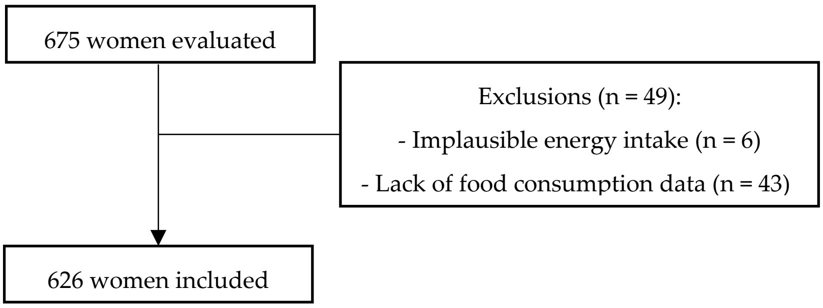

TABLE S1 - Mean maternal energy percentual consumption by food processing groups during pregnancy

**Food processing group**	**% of total daily caloric value**
In natura or minimally processed	50.01
Processed	19.43
Ultra-processed	30.56
